# World Statistics Drive Learning of Cerebellar Internal Models in Adaptive Feedback Control: A Case Study Using the Optokinetic Reflex

**DOI:** 10.3389/fnsys.2020.00011

**Published:** 2020-03-25

**Authors:** Sean R. Anderson, John Porrill, Paul Dean

**Affiliations:** ^1^Department of Automatic Control and Systems Engineering, University of Sheffield, Sheffield, United Kingdom; ^2^Department of Psychology, University of Sheffield, Sheffield, United Kingdom

**Keywords:** cerebellar, computational model, optokinetic (OKN) system, world statistics, adaptive filter

## Abstract

The cerebellum is widely implicated in having an important role in adaptive motor control. Many of the computational studies on cerebellar motor control to date have focused on the associated architecture and learning algorithms in an effort to further understand cerebellar function. In this paper we switch focus to the signals driving cerebellar adaptation that arise through different motor behavior. To do this, we investigate computationally the contribution of the cerebellum to the optokinetic reflex (OKR), a visual feedback control scheme for image stabilization. We develop a computational model of the adaptation of the cerebellar response to the world velocity signals that excite the OKR (where world velocity signals are used to emulate head velocity signals when studying the OKR in head-fixed experimental laboratory conditions). The results show that the filter learnt by the cerebellar model is highly dependent on the power spectrum of the colored noise world velocity excitation signal. Thus, the key finding here is that the cerebellar filter is determined by the statistics of the OKR excitation signal.

## Introduction

Eye movements have been used extensively to investigate the functions of the cerebellum in motor control (Carpenter, 1988; Büttner-ennever, 2006). They are mechanically much simpler than movements of multi-joint limbs, and the neural circuitry underlying their control is corresponding less complicated (Robinson, 1986). Historically one type of eye movement has been of particular interest, namely that made in response to unexpected rotations of the head. The eyes rotate in the opposite direction to the head so as to stabilize the direction of gaze in space, thus minimizing the extent of whole image movement over the retina (retinal slip) and consequent loss of visual information. Since the reflex is driven by information regarding head movement provided by the vestibular system it is termed the vestibulo-ocular reflex (VOR). One of its key features is that its gain can be altered (VOR adaptation), and Ito (1970) first appreciated the importance of the cerebellum on mediating this adaptation, proposing the flocculus as the region of the cerebellum involved. Subsequent experimental and modeling work on the role of the cerebellum in VOR adaptation continues to the present (e.g., Inagaki and Hirata, 2017; Voges et al., 2017; Luque et al., 2019).

**Figure 1:**
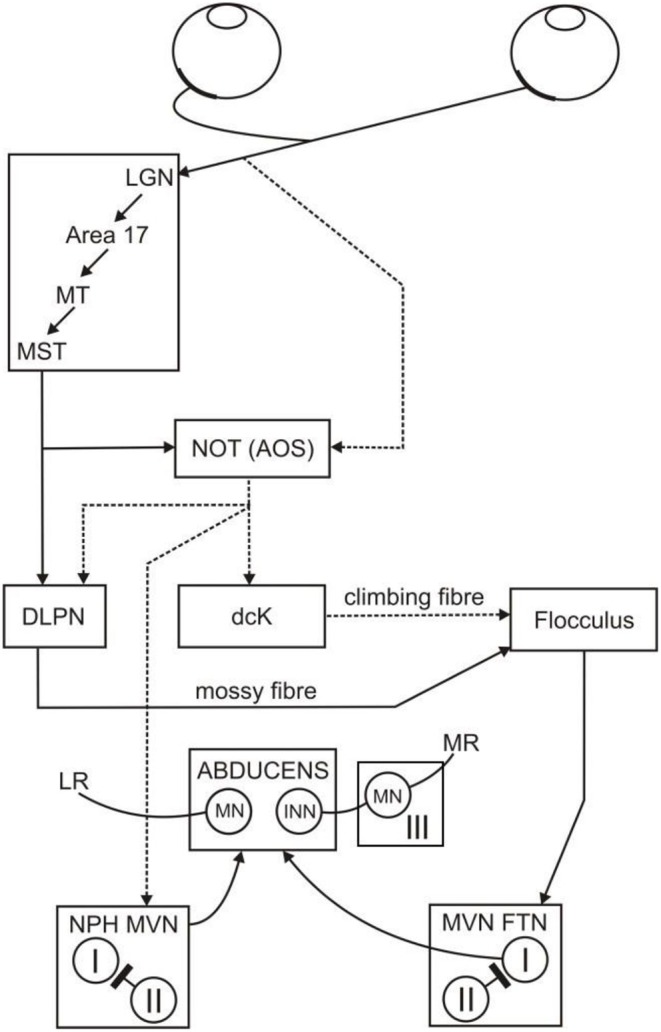
Neural substrate of the horizontal OKR. The retinal slip signal that drives the OKR arrives at the flocculus via climbing fibers (NOT, dcK) and mossy fibers (MST, NOT, DLPN, the direct pathway). The retinal slip signal is also transmitted to the brainstem (NPH, MVN, the indirect pathway). The direct and indirect pathways both project to the abducens nucleus and from there combine to drive movements of the eye in order to stabilize the visual image. From Figure 12 of Mustari et al. (1994). I and II, type 1 and 2 neurons in the vestibular nucleus; III, oculomotor nucleus; AOS, accessory optic system; dcK, dorsal cap of Kooy of the inferior olive; DLPN, dorsolateral pontine nucleus; FTN, floccular target neurons; INN, internuclear neurons; LR, lateral rectus; LGN, lateral geniculate nucleus; MN, motor neurons; MR, medial rectus; MST, middle superior temporal gyrus of cerebral cortex; MT, middle temporal cortex; MVN, medial vestibular nucleus; NPH, nucleus prepositus hypoglossi; NOT, nucleus of the optic tract.

Since movements of the eyes have no effect on head position, the VOR is functionally a *feedforward* control scheme, and VOR adaptation provides a mechanism that enables the scheme to be calibrated. But there is also a *feedback* control scheme for stabilizing gaze, which uses retinal slip itself to drive counter-rotatory eye movements, and is termed the optokinetic reflex (OKR): the neural substrate of the horizontal OKR is shown in Figure 1. This reflex works cooperatively with the VOR to stabilize gaze (Carpenter, 1988). The need for two control schemes (VOR and OKR) to perform one task (image stabilization) is explained by the respective operational range of the VOR and OKR: the VOR does not work effectively at low frequencies of head movement, due to poor sensing of the head velocity at those frequencies by the semicircular canals of the vestibular system. In contrast, the OKR does not function well at high frequencies of head movement because the retinal slip is delayed (by ~100 ms) due to visual processing time (Robinson, 1987). Hence, the VOR and OKR combine to stabilize the full field visual image across a wider frequency spectrum than might be accomplished individually (Paige, 1983; Godaux and Vanderkelen, 1984; Boyle et al., 1985; Schweigart et al., 1997).

Here we model how OKR performance is improved by the contribution of the cerebellar flocculus, using an architecture based on the detailed descriptions available of the relevant neural circuitry (Fuchs and Mustari, 1993; Mustari et al., 1994). The flocculus itself is represented by the adaptive-filter model of the cerebellar cortical microcircuitry, proposed by Fujita (1982) as a development of the original Marr-Albus framework to allow direct processing of temporally varying inputs, and consequent representation of systems in which current output depends on input history. Adaptive-filter models have subsequently used extensively in system-level modeling of cerebellar function (Dean and Porrill, 2010).

We focus our analysis of the adaptive OKR model on the retinal slip signal which is used to excite the feedback control loop. The term “excite” is used in the systems engineering sense of an *excitation signal*, which refers to the input signal that causes the system to generate an output. Excitation signals can be divided into two classes: predictable and non-predictable. An example of a predictable signal is a single frequency sine wave, whilst a non-predictable signal is colored noise. Our analysis on these two classes of signal reveals the extreme dependence of cerebellar learning on the predictability of the excitation signal, in other words on the statistics of the visual world.

The OKR is an exemplar problem of adaptive feedback control. Hence, understanding how the cerebellum adapts to optimize eye movements in the OKR is potentially of great importance for understanding cerebellar involvement in feedback control throughout the nervous system.

## Methods

The basic circuitry underlying the primate OKR has been described by Mustari et al. (1994). The box labeled flocculus in this figure refers to those microzones in the flocculus and ventral paraflocculus that are concerned with conjugate horizontal eye movements (i.e., rotations around a vertical axis). These microzones, and their connectivity, are described in detail by Voogd and Barmack (2006).

**Figure 2:**
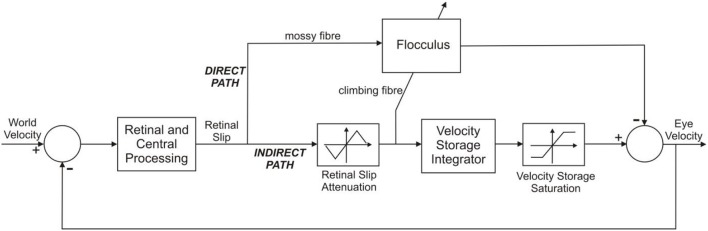
Systems level model of the OKR. The OKR functions as an adaptive feedback control loop. World velocity excites the control scheme; error between world velocity and eye velocity drives the controllers of which there are two: (i) In the indirect pathway there is a fixed controller (located in the brainstem), known as the velocity storage integrator. (ii) The floccular region of the cerebellum is in the direct pathway of the OKR. The flocculus provides adaptive compensation of the OKR. The indirect pathway has static non-linearities that affect performance at velocities higher than ~50 deg/s. The other important feature relating to feedback control is that the error signal, retinal slip, is delayed in visual processing by about 100 ms. Adapted from Figure 10 of WRC83.

Here we model this connectivity using the simplified architecture illustrated in Figure 2, which is based on the previous model of Waespe et al. (1983) (subsequently referred to as WRC83), with the vestibular pathways of that model omitted because in the experimental conditions modeled here the head is fixed so no vestibular signals are involved. This architecture is also consistent with other models of the OKR, for instance that of Buizza and Schmid (1982). We focused on linear analysis of the system, and therefore ignored the effects of the static non-linearities in the indirect pathway, activated at velocities >50 deg/s in primate WRC83.

The input to the feedback loop shown in Figure 2 is “world velocity,” appropriate for experimental studies that investigate the OKR under head fixed conditions. Here the subject is typically sat, head fixed, inside a rotating drum, so that it appears as if the world itself were moving. In non-experimental conditions the OKR would be excited by head velocity. However, head velocity is fixed to zero in experimental conditions when studying the OKR, in order to keep the eye movement response independent of the VOR. The world velocity signal is transformed by retinal and central processing into a retinal slip signal that is utilized by two main neural pathways, one the “direct” pathway through the cerebellum, the other the “indirect” pathway through the brainstem. The indirect pathway incorporates a velocity storage unit which integrates the retinal slip signal and provides a slow response component to the OKR, revealed when the flocculus is inactivated WRC83. The cerebellar contribution to the OKR is a rapid rise in eye velocity early in the response, modeled by a filter with a fast time constant, which is the case here.

The flocculus itself is modeled as an adaptive filter, which represents plasticity at the parallel fiber/Purkinje cell (PF/PC) synapse by an anti-Hebbian learning rule (Sejnowski, 1977; Fujita, 1982): synaptic efficacy changes in response to correlation in PF and CF firing. Positive correlation in PF/CF firing results in positive weight change and negative correlation results in negative weight change. Hence, LTP and LTD are modeled using this learning rule, both of which occur at the PF/PC synapse (Ito, 1989; Coesmans et al., 2004) (further details below).

**Figure 3:**
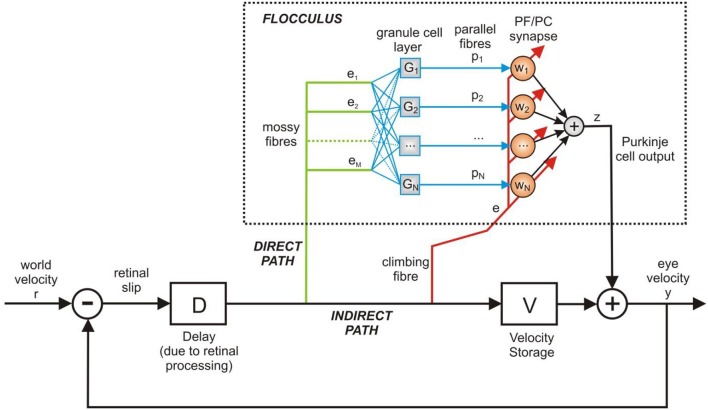
Adaptive filter model of the cerebellum in the OKR control loop. The adaptive filter models the signal processing in the floccular region of the cerebellum. Here the adaptive filter model is inserted in the OKR model shown in Figure 2. The velocity storage is modeled here by a linear 1st order transfer function. The output of cerebellum and brainstem are assumed to sum together to produce the motor command, which here is equivalent to eye velocity.

The architecture in Figure 3 can be described in terms of three distinct functional elements using Laplace transforms: (i) a time-delay operator *D*(*s*) = *e*^−*ds*^, of delay *d* seconds, (ii) the floccular region of the cerebellum *C*(*s*) and (iii) the velocity storage unit *V*(*s*). We can form the closed loop description of the OKR model in Figure 3, which is

(1) $$\begin{array}{l} \begin{array}{c} Y\left(s\right)=\frac{D\left(s\right)\left[C\left(s\right)+V\left(s\right)\right]}{1+D\left(s\right)\left[C\left(s\right)+V\left(s\right)\right]}R\left(s\right) \end{array} \end{array}$$

where *Y*(*s*) is the Laplace transform of the eye velocity *y*(*t*) and *R*(*s*) is the Laplace transform of the world, or head, velocity *r*(*t*) (note that world velocity excites the OKR in experimental setups where the head is fixed and the world rotates, whilst head velocity excites the OKR in natural conditions where the head is free to move and the world is fixed).

### Model of the Velocity Storage Unit

The velocity storage unit in Figure 3 was modeled as a first order transfer function *V*(*s*), of the form

(2) $$\begin{array}{l} \begin{array}{c} V\left(s\right)=\frac{K_{v}}{T_{v}s+1} \end{array} \end{array}$$

where *K*_*v*_ is a gain term and *T*_*v*_ is a time constant.

The gain and time constant of *V*(*s*) were estimated by grid search, minimizing the sum-of-squared error (SSE) fit to step response data obtained after floccular removal in primate (flocculectomy) from WRC83, Figure 11D. Note that the closed loop OKR system given in (1), for the flocculectomy condition where *C*(*s*) = 0, reduces to

(3) $$\begin{array}{l} \begin{array}{c} Y\left(s\right)=\frac{D\left(s\right)V\left(s\right)}{1+D\left(s\right)V\left(s\right)}R\left(s\right)=\frac{e^{-ds}K_{v}}{T_{v}s+e^{-ds}K_{v}+1}R\left(s\right) \end{array} \end{array}$$

where the closed loop transfer function has the steady-state gain *K*_*ss*_ = *K*_*v*_/(*K*_*v*_ + 1). This means that for a plausible steady-state OKR flocculectomy gain 0.9 ≤ *K*_*ss*_ ≤ 0.95, the velocity storage gain is approximately 9 ≤ *K*_*v*_ ≤ 20. Therefore, the value of *K*_*v*_ was estimated by performing a grid search over this range, 9 ≤ *K*_*v*_ ≤ 20, in steps of 0.5. The time constant value *T*_*v*_, was estimated by searching across the range 100 ≤ *T*_*v*_ ≤ 300 s, in steps of 10 s.

### Adaptive Filter Model of the Cerebellum

The adaptive filter model of the cerebellum used here is equivalent to the original implementation by Fujita (1982). Hence, for computational expedience we represent a single Purkinje cell, but mathematically this is equivalent to an arbitrary number of Purkinje cells, under the assumption that their outputs sum linearly.

The mossy fiber input to the cerebellum is processed by the granule cell layer, which is represented by a bank of *N* linear filters *G*_*i*_(*s*), for *i* = 1, …, *N* (see Figure 3). The exact nature of the filters *G*_*i*_(*s*) is not critical: the function of *G*_*i*_(*s*) is to act analogously to a tap-delay line, which can be achieved by using alpha-functions that have well-spaced time constants, so that

(4) $$\begin{array}{l} \begin{array}{c} G_{i}\left(s\right)=\frac{K_{G}^{\left(i\right)}}{{\left(T_{G}^{\left(i\right)}s+1\right)}^{2}}\text{ for }i=1,\ldots ,N \end{array} \end{array}$$

Where $T_{G}^{\left(i\right)}$ is the alpha-function time constant and $K_{G}=1/{\left(T_{G}^{\left(i\right)}\right)}^{2}$.

We assume that mossy fibers transmit the OKR error signal *e*(*t*), with Laplace transform *E*(*s*), which is processed by the granule cell layer represented by the filter bank *G*_*i*_(*s*), to produce the PF signal *P*_*i*_(*s*), that is

(5) $$\begin{array}{l} \begin{array}{c} P_{i}\left(s\right)=G_{i}\left(s\right)E\left(s\right)\text{ for }i=1,\ldots ,N \end{array} \end{array}$$

The PF signals, in the context of the OKR, can therefore be interpreted as a set of retinal slip signals, delayed by varying amounts by the granule cell layer.

The cerebellar model output, *Z*(*s*), is the sum of weighted PF signals,

(6) $$\begin{array}{l} \begin{array}{c} Z\left(s\right)=\overset{N}{\underset{i\;=\;1}{\sum }}w_{i}P_{i}\left(s\right)\text{ for }i=1,\ldots ,N \end{array} \end{array}$$

where the plasticity of the PF/PC weights *w*_*i*_ is modeled by an anti-Hebbian learning rule discussed below. The cerebellar filter *C*(*s*) is now conveniently described from mossy fiber input to PC output by the expression

(7) $$\begin{array}{l} \begin{array}{c} C\left(s\right)=\overset{N}{\underset{i\;=\;1}{\sum }}w_{i}G_{i}\left(s\right)\text{ for }i=1,\ldots ,N \end{array} \end{array}$$

The expression for *C*(*s*) in Equation (7) is a more functionally realistic representation of the cerebellum in comparison to the proportional gain model in WRC83 because (i) it can represent varying gain across the frequency spectrum and (ii) it facilitates modeling and analyzing plasticity at the PF/PC synapse.

### Learning Rule at the PF/PC Synapse

The adaptation at the PF/PC synapse is modeled by the correlation of delayed PF signal and CF signal,

(8) $$\begin{array}{l} \begin{array}{c} \Delta w_{i}=-\beta \langle e\left(t\right)p_{i}^{\left(d\right)}\left(t\right)\rangle \end{array} \end{array}$$

where *w*_*i*_ is the i^th^ weight in the cerebellar filter, β is a constant term that adjusts the learning rate, *e*(*t*) is the error signal on the CF and $p_{i}^{\left(d\right)}\left(t\right)$ is the i^th^ PF signal, whilst the superscript *d* indicates that the PF signal is delayed by *d* seconds in the learning rule. It has been found in a modeling study of the ocular following response that a delay was required for stable adaptation in the PF/PC synapse learning rule (Yamamoto et al., 2002) and that is also the case here as using an un-delayed PF signal in the learning rule led to unstable adaptation.

We implemented the delay in the learning rule by an eligibility trace. The eligibility trace is a biologically plausible representation of a delay mechanism occurring in synaptic plasticity (Kettner et al., 1997; Wang et al., 2000). The eligibility trace was dynamically represented here by an α-function *L*(*s*), where the time constant was set to the delay in retinal slip visual processing,

(9) $$\begin{array}{l} \begin{array}{c} L\left(s\right)=\frac{1}{{\left(1+ds\right)}^{2}} \end{array} \end{array}$$

The input to the eligibility trace is the parallel fiber signal *p*_*i*_(*t*) and the output is a filtered version of *p*_*i*_(*t*) which peaks at *d* seconds,

(10) $$\begin{array}{l} \begin{array}{c} P_{i}^{\left(d\right)}\left(s\right)=L\left(s\right)P_{i}\left(s\right) \end{array} \end{array}$$

where $P_{i}^{\left(d\right)}\left(s\right)$ and *P*_*i*_(*s*) are the Laplace transforms of $p_{i}^{\left(d\right)}\left(t\right)$ and *p*_*i*_(*t*), respectively.

### OKR Excitation by Predictable and Non-predictable Signals

The world velocity signal *r*(*t*) was used to excite the OKR model. The signal *r*(*t*) can be defined as either a predictable signal such as a sine wave, or non-predictable signal such as colored noise **or white noise**. We suggest that during the natural operation of the OKR the excitation signal is well-represented by colored noise, consistent with head velocity being stochastic and hence unpredictable at any point in time, but characterized by fixed statistical properties and structured as suggested by the data in Carriot et al. (2017). Sine waves are often used in experimental investigations of the OKR, as well as computational studies and were therefore also investigated here.

For the colored noise world velocity signal *r*(*t*), the power spectral density was set to

(11) $$\begin{array}{l} \begin{array}{c} S_{RR}\left(f\right)=\frac{b}{f^{a}} \end{array} \end{array}$$

where *f* is frequency in Hertz, *a* is the spectral exponent and *b* is the constant of proportionality. Note that spectral exponent *a* = 0 corresponds to white noise, *a* = 1 corresponds to pink noise, and *a* = 2 corresponds to red noise. Here we only considered a value of *a* = 1.2, which has been shown to plausibly represent power in head yaw movements (Carriot et al., 2017). The scaling parameter *b* was adjusted by numerical simulation to fit behavioral data (see Results).

### Simulation Details

To simulate the computational model of the OKR depicted in Figure 3, each of the transfer functions defined above in the Laplace-domain, time-delay *D*(*s*), velocity storage *V*(*s*), granule layer basis functions *G*_*j*_(*s*) and eligibility trace *L*(*s*), were parametrised using the values in Table 1 and defined in Matlab for simulation. The transfer functions were discretised using a zero-order hold at a sample time of 0.1 s to produce the Z-transform equivalents for simulation in discrete-time: $V\left(z\right)=\overset{\infty }{\underset{n=0}{\sum }}v\left(n\right)z^{-n}$, $G_{j}\left(z\right)=\overset{\infty }{\underset{n=0}{\sum }}g_{j}\left(n\right)z^{-n}$, $L\left(z\right)=\overset{\infty }{\underset{n=0}{\sum }}l\left(n\right)z^{-n}$ and $D\left(z\right)=z^{-n_{d}}$ (i.e., a pure delay of *n*_*d*_ time-steps).

**Table 1:** Parameter values used in the computational simulations.

**Parameter**	**Symbol**	**Value**
Velocity storage time constant	*T*_*V*_	230 s
Velocity storage gain	*K*_*V*_	13.5
Number of cerebellar filter weights	*N*	5
Cerebellar filter alpha function time constants	$T_{G}^{\left(1,\ldots ,5\right)}$	0.01, 0.02, 0.1, 0.2, 0.5 s
Cerebellar filter learning rate	β	0.001
Initial cerebellar filter weight values	*w*_1, …, 5_(0)	0
Retinal slip delay	*d*	0.1 s

The colored noise world velocity signal *r*(*t*) was created for the discrete-time simulations by generating samples of a white noise signal, then taking the discrete Fourier transform of this signal, applying the *b*/*f*^*a*^ transformation in the frequency-domain, then taking the inverse discrete Fourier transform to finally obtain the sampled colored noise world velocity signal *r*(*k*).

The algorithm for evaluating the OKR model output is as follows: at a sample instant denoted by *k* sequentially evaluate

$$\begin{array}{l} e_{d}\left(k\right)=e\left(k-n_{d}\right)\;\left(\text{delayed retinal slip}\right)\\ \;x\left(k\right)=\sum_{n\;=\;0}^{\infty }v\left(n\right)e_{d}\left(k-n\right)\;\left(\text{velocity storage output}\right)\\ \;z\left(k\right)=\sum_{n\;=\;0}^{\infty }c\left(n,k\right)e_{d}\left(k-n\right)\;\left(\text{cerebellar output}\right)\\ \;y\left(k\right)=z\left(k\right)+x\left(k\right)\;\left(\text{eye velocity output}\right)\\ \;e\left(k\right)=r\left(k\right)-y\left(k\right)\;\left(\text{retinal slip}\right) \end{array}$$

where the cerebellar filter $C\left(z,k\right)=\overset{\infty }{\underset{n\;=\;0}{\sum }}c\left(n,k\right)z^{-n}$ is defined by the adaptation of the filter weights, using the learning rule in (7), where

$$\begin{array}{l} \;p_{j}\left(k\right)=\sum_{n\;=\;0}^{\infty }g_{j}\left(n\right)e_{d}\left(k-n\right)\;\left(\text{PF signal}\right)\\ \;p_{j}^{\left(d\right)}\left(k\right)=\sum_{n\;=\;0}^{\infty }l\left(n\right)p_{j}\left(k-n\right)\;(\text{PF signal delayed by}\\ \text{ eligibility trace})\\ \;\Delta w_{j}\left(k\right)=-\beta e_{d}\left(k\right)p_{j}^{\left(d\right)}\left(k\right)\;(\text{PF}/\text{PCcerebellar}\\ \text{ weight adaptation})\\ \;w_{j}\left(k+1\right)=w_{j}\left(k\right)+\Delta w_{j}\left(k\right)\;(\text{updated cerebellar}\\ \text{ filter weights})\\ C\left(z,k+1\right)=\sum_{j\;=\;1}^{N}w_{j}\left(k+1\right)G_{j}\left(z\right)\;(\text{updated cerebellar}\\ \text{ filter}) \end{array}$$

To improve stability of the weight adaptation rule the cerebellar weights were updated in a batch mode of 10,000 samples (corresponding to 1,000 s of data), over 2,000 batch iterations. The weight change across batches was monitored to ensure convergence.

## Results

### Adaptive Model Describes the OKR Step Response in Primate

**Figure 4:**
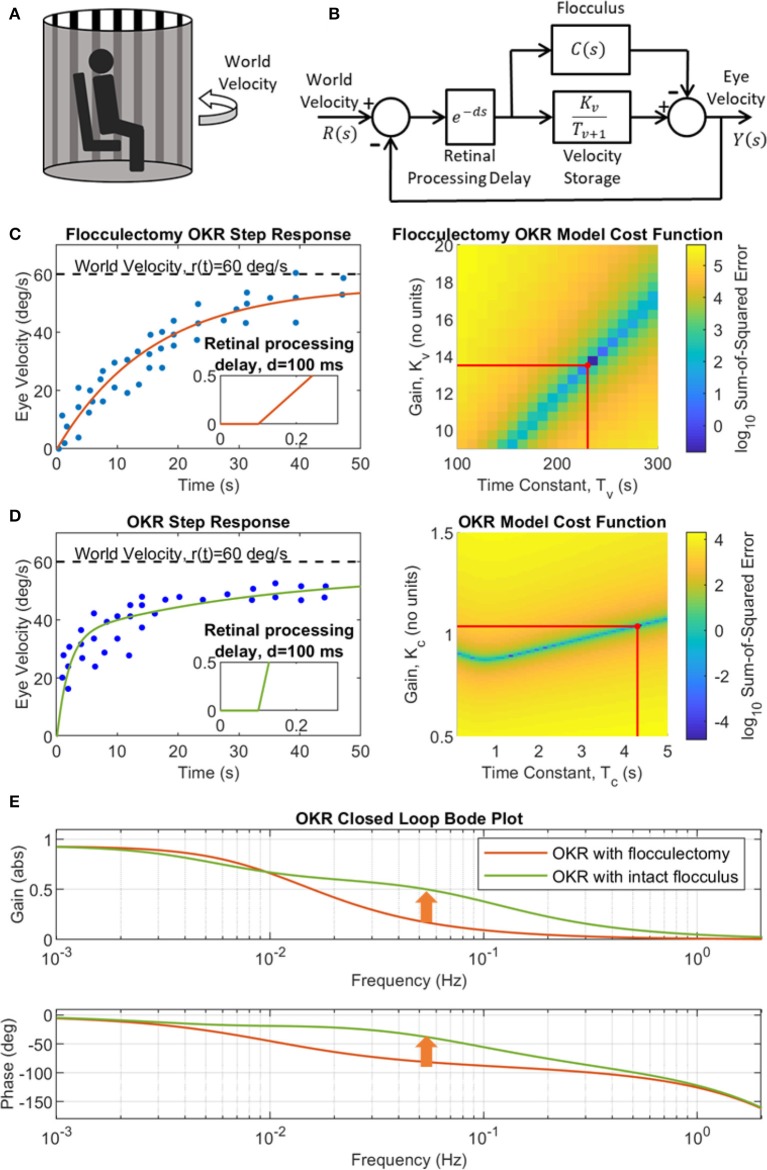
A simple linear model of the OKR for flocculectomy and intact flocculus conditions. **(A)** Illustration of a typical OKR experimental setup, where the subject sits, head-fixed, in a rotating drum that has alternating vertical black and white stripes on the interior. **(B)** Simplified linear OKR feedback control loop with floccular region of the cerebellum modeled by a fixed transfer function **C**(**s**)**=K**_**C**_**/(T**_**C**_**s+1)**. **(C)** Identification of the velocity storage transfer function. Left panel: OKR flocculectomy step response (*C*(*s*) = 0) to a step of 60 deg/s where the data (dots) were extracted from Waespe et al. (1983), Figure 11D, with simulation of the estimated best model fit (solid line) in closed loop, where *T*_*v*_ = 230 s and *K*_*v*_ = 13.5. Right panel: Grid search cost function (color map) for the optimal velocity storage parameters (red lines), *K*_*v*_ and *T*_*v*_. **(D)** Identification of the fixed cerebellar filter modeled as a first order transfer function, *C*(*s*) = *K*_*C*_/(*T*_*C*_*s*+1). Left panel: OKR step response with intact cerebellum where the data (dots) were extracted from WRC83, Figure 11B, with simulation of the feeback loop in **(B)** with best cerebellar model fit (solid line) where *T*_*C*_ = 4.3 s and gain *K*_*C*_ = 1.04. Right panel: Grid search cost function (color map) for the optimal cerebellar filter parameters (red lines), *K*_*C*_ and *T*_*C*_. **(E)** OKR closed loop Bode plot for both flocculectomy and intact flocculus conditions derived from the model in panel B, using the transfer functions identified in **(C,D)**. The cerebellum contributes a rise in gain toward one, and phase toward zero, in the region of 0.1 Hz (indicated by the arrows), which improves control performance.

In previous non-adaptive models of the OKR their parameters were specifically tuned to produce the eye movements observed experimentally. Here such tuning was used only for the time constant and gain of the velocity-storage component (Equation 2) and to illustrate the contribution of the cerebellum, in the time-domain and the frequency-domain (Figure 4). The experimental setup that the model simulations emulate is shown in Figure 4A, with the corresponding feedback control loop in Figure 4B. The response of the OKR model without cerebellar contribution (flocculectomy condition) to a velocity step input of 60 deg/s, compared with experimental observations from WRC83 of OKR performance after floccular removal is shown in Figure 4C. A reasonable fit has been obtained with a time constant of 230 s and gain of 13.5, producing a rise time of ~20 s. The fit shown in Figure 4C suggests that the first order filter characterization of the velocity-storage element is a reasonable approximation. The contribution of the cerebellum is illustrated in Figure 4D using a fixed first order filter. This linear modeling of the OKR using transfer functions enables the computation of a Bode plot to compare the flocculectomy and intact characteristics of the OKR in the frequency-domain (Figure 4E).

**Figure 5:**
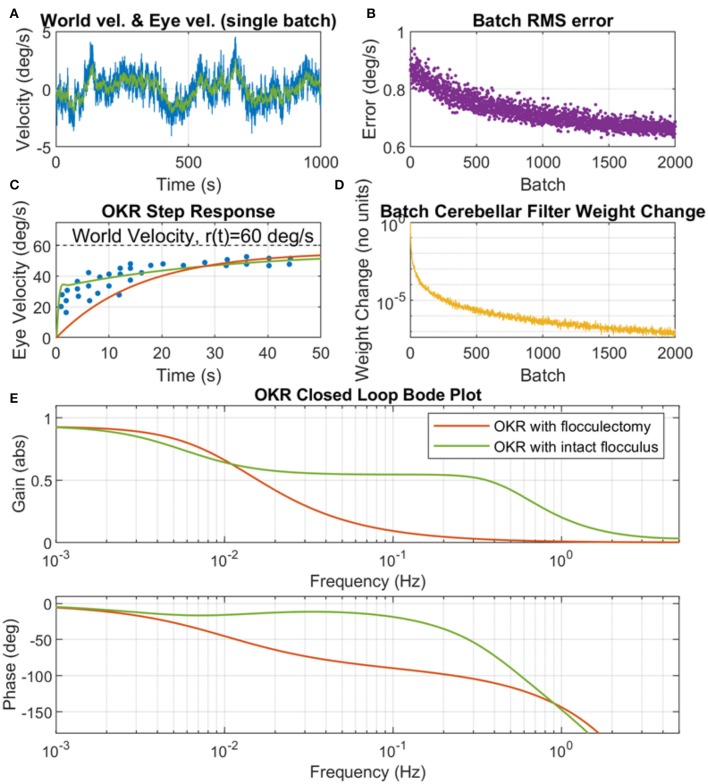
Cerebellar adaptation in the OKR driven by a colored noise world velocity signal. **(A)** Example of a single batch of world velocity (blue line) and eye velocity (green line) simulation data, where world velocity has a spectral exponent of *a* = 1.2 and scaling parameter b = 0.017. **(B)** Batch root mean square (RMS) error (where error is the difference between world velocity and eye velocity). **(C)** OKR step response where experimental data (dots) is from WRC83, Figure 11B, OKR model step response with intact flocculus (solid green line) is from training on 2,000 batches of simulation data with colored noise world velocity signal, and OKR flocculectomy model response (solid red line) is without a cerebellar contribution. **(D)** Batch cerebellar filter weight change demonstrating convergence of the adaptive cerebellar filter in the OKR control loop. The adaptation rule is stochastic hence does not go to zero but note the log scale, which shows very small numerical change in the weights by batch 2,000. **(E)** OKR closed loop Bode plot for both flocculectomy and intact flocculus conditions, where the Bode plots are obtained from the same models as the step responses in **(C)**.

To explore the contribution of the flocculus to OKR performance under learning conditions, the adaptive-filter model of the cerebellum was initialized to zero, then learning was driven by exciting the OKR system with a colored noise world-velocity signal, which indirectly caused parameter adaptation as a result of the learning rule describing plasticity at the PF/PC synapse. We found that when the colored noise world velocity signal spectral exponent was set to *a* = 1.2, and the scaling parameter of the noise set to *b* = 0.017, the adaptive OKR model converged to a feedback control scheme that produced an OKR step response closely resembling behavioral data (Figure 5). The adaptive filter model of the flocculus modified the OKR dynamics by causing a rapid increase in velocity early in the step response compared to the flocculectomy condition (Figure 5C) and correspondingly raising the closed loop OKR gain in the region of 0.1 Hz (Figure 5E).

### The Learned Cerebellar Filter Depends on the Statistics of the Excitation Signal

**Figure 6:**
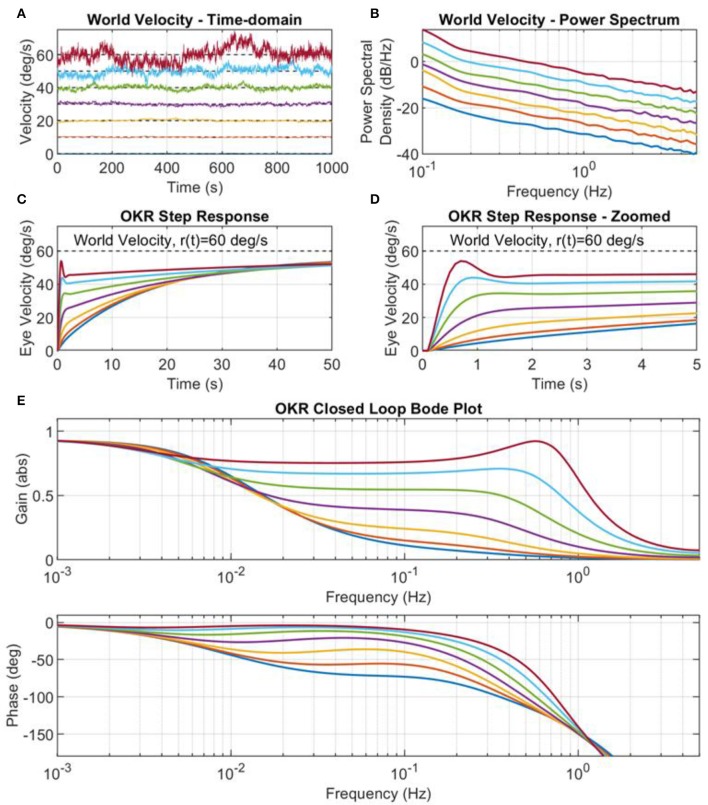
World velocity colored noise statistics drives adaptation of the cerebellar filter. **(A)** Examples of world velocity colored noise signals used to drive adaptation of the cerebellar filter in the OKR control loop (note that each signal is successively offset for plotting by 10 deg/s for clarity, and that each signal had zero mean). Each signal has the same spectral exponent *a* = 1.2, but different scaling parameters *b*. The power spectrum scaling parameters, *b*, were obtained from testing 10 values log-spaced in the amplitude range *k*_*b*_ ∈ [0.01, 0.1], where $b=k_{b}^{2}$, but only the results from values two to eight are shown here, i.e., where *b* values were set to 0.0003, 0.0008, 0.0022, 0.0060, 0.0167, 0.0464, 0.1292. **(B)** Power spectrum of the world velocity signals shown in **(A)**. **(C)** Step response of the closed loop OKR system after 2,000 batches of cerebellar training updates. **(D)** Step response of the closed loop OKR system after 2,000 batches of cerebellar training updates, zoomed on the time axis. **(E)** OKR closed loop Bode plots corresponding to the OKR step responses in **(C,D)**.

The adaptation of the OKR is caused by the retinal slip error. The characteristics of this error signal are directly dependent on the statistics of the world velocity excitation signal. For instance, we have shown in Figure 5 that when the scaling parameter and spectral exponent of the colored noise excitation signal are appropriately tuned, the excitation signal produces learning that converges to a system that reproduces behavioral OKR data. The implication of this result is that the excitation signal can be manipulated to cause a variety of different OKR responses. Here we simulated the adaptive OKR model with colored noise signal in a number of experimental trials, altering both the scaling parameter, *b* (Figure 6), and the spectral exponent, *a* (results not shown), to observe the effect on the OKR dynamics.

We systematically varied the scaling parameter, *b*, of the colored noise world velocity signal to produce varying power levels of excitation of the OKR model (Figures 6A,B). The resulting step responses of the OKR model are shown in Figures 6C,D. The early rapid rise in velocity, due to the cerebellar filter, increases in amplitude as the scaling parameter *b* increases, even leading to overshoot and oscillation in the eye velocity response (Figures 6C,D). The step responses and Bode plots of the OKR shown in Figures 6C–E are significantly different from each other: the implication of this result is that world velocity statistics have a strong influence on the resultant learned characteristics of the OKR.

### The OKR Performance Improves When Excited by Simple Predictable Signals

The control of eye movements is often studied by exciting the oculomotor system with a single frequency sine wave. This would correspond to causing the full field image to oscillate at a single frequency in the case of the OKR (Paige, 1983; Boyle et al., 1985) or a target in the case of smooth pursuit (Deno et al., 1989). Studies of the OKR and smooth pursuit systems have revealed that the control performance in terms of gain and phase improve when excited by predictable signals (Wyatt and Pola, 1988; Deno et al., 1989). Here we investigated this phenomenon by exciting the adaptive OKR model with both predictable and non-predictable signals.

The predictable excitation signals in the simulation model were designed to emulate single frequency sine wave excitation of the OKR often used in experimental setups (Paige, 1983; Boyle et al., 1985). The OKR model was initialized to a “natural” state before each trial: rather than setting the adaptive filter weights to zero, we initialized the weights using the trained the OKR model after adaptation driven by the colored noise excitation signal with scaling parameter *b* = 0.017 and spectral exponent *a* = 1.2, which reproduced behavioral data shown in Figure 5. The OKR model was then excited in each sine wave trial for just 600 s (sequential adaptation was used in the cerebellar filter not batch adaptation). To a certain extent this setup emulates the somewhat artificial experimental conditions where a normal subject undergoes excitation of the OKR using single frequency sine waves.

**Figure 7:**
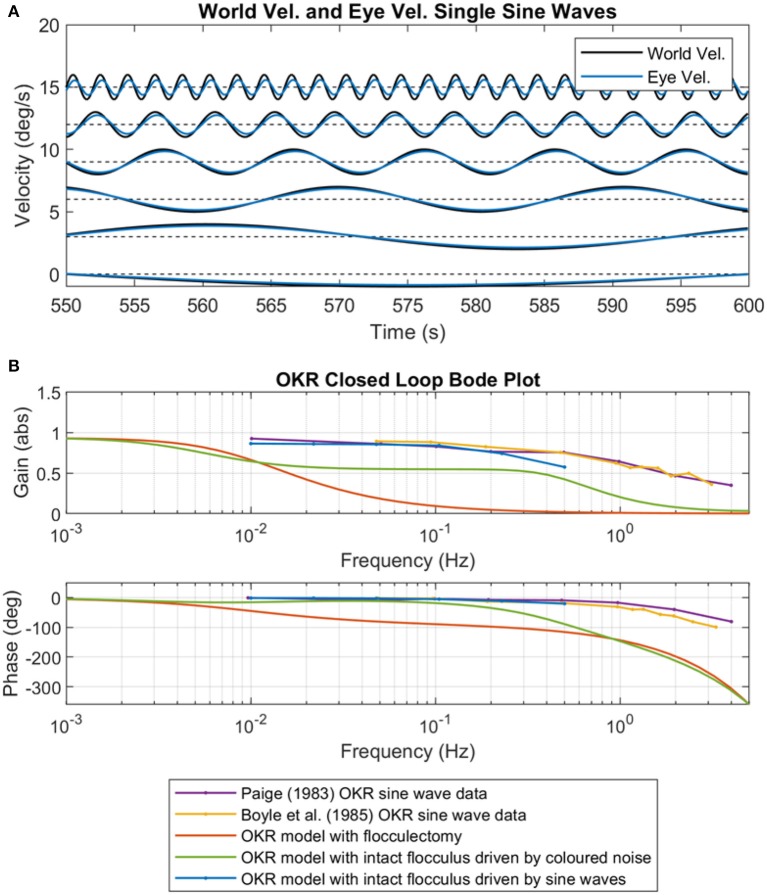
OKR performance after training on predictable and non-predictable signals. **(A)** Predictable sine waves signals (frequencies were six log-spaced values from 0.01 to 0.5 Hz) used as the world velocity signal to excite the OKR control loop. Also shown are the corresponding eye velocity output signals after cerebellar adaptation, from 600 s of online training (non-batch), zoomed to the final 50 s of adaptation. **(B)** OKR closed loop Bode plots from OKR sinusoidal experimental data (Paige, 1983; Boyle et al., 1985), the original OKR flocculectomy model from this paper, the OKR model with intact cerebellum, and the Bode plot reconstructed from the individual sinusoidal excitations shown in **(A)**.

The closed loop performance of the OKR model greatly improved, in terms of closed loop gain and phase, when exciting the system with single frequency sine waves (a predictable signal) rather than colored noise (an unpredictable signal) (Figures 7A,B). The implication of this result is that cerebellar model is able to rapidly learn to compensate for the specific and fixed characteristics of the predictable sine wave signal. It is also apparent that the modeling results are qualitatively similar to the experimental results from Paige (1983) and Boyle et al. (1985) (Figure 7B), which suggests that experimental results reporting OKR characteristics using single frequency sine waves do not reflect “normal” operation.

## Discussion

### Consistency With Experimental Data

#### Predictability and the OKR

Improvements in OKR performance related to stimulus predictability were initially described by Yasui and Young (1984) and Wyatt and Pola (1988) in humans, and subsequently in a wide range of species (Miki et al., 2018). Optokinetic adaptation, in which simple exposure to a sinusoidally moving wide-field stimulus increases OKR gain, is a well-studied example (e.g., Inoshita and Hirano, 2018).

#### Delay: Stable and Convergent Adaptation Requires a Delayed PF Signal in the Learning Rule

The PF signal requires a delay in the learning rule to ensure that learning is stable and convergent. It is well-known from the systems engineering literature that stability in correlation based learning rules is dependent on the correlating signals being within +/−90 degrees of the correct phase with each other (Vaudrey et al., 2003). We observed instability in the learning rule when the PF signal was not delayed, similarly to Yamamoto et al. (2002). Evidence for a delayed error signal (~120 ms) has come from studies of cerebellar long-term depression (Suvrathan et al., 2016; Suvrathan and Raymond, 2018), which show that this delay occurs in the flocculus that deals with the OKR, while other delays are found elsewhere in the cerebellum.

### Computational Framework

It has long been recognized that while the structure of cerebellar cortex is relatively uniform, different regions of cortex have different connections to external structures such as the deep cerebellar nuclei and inferior olive. This has given rise to the “chip” metaphor (e.g., Ito, 1997; Porrill et al., 2013), in which the cerebellar cortex is cast as a set of identical chips that can be used for a wide variety of purposes. In this context there are two aspects to a cerebellar model, one its representation of the microcircuit, and the second its external connectivity.

#### Microcircuit Model

Early modeling studies of the OKR in rabbit (Collewijn, 1969, 1972), cat (Buizza and Schmid, 1982; Gillis et al., 1984) and primate (Buizza and Schmid, 1982; Waespe et al., 1983) have represented the cerebellar OKR function as a single fixed gain. Here we use the much more powerful adaptive-filter model of the cerebellar microcircuit. First proposed by Fujita (1982), this model extends the original Marr-Albus framework to cope with dynamic time-varying signals, and is thus very well-suited to the construction of internal models of dynamic processes (Dean et al., 2010; Porrill et al., 2013).

#### Connectivity

The floccular connectivity illustrated in Figure 1 is simplified in two main ways. First, as described in Methods, the box labeled flocculus in this figure refers to those microzones in the flocculus and ventral paraflocculus that are concerned with conjugate horizontal eye movements (i.e., rotations around a vertical axis). These microzones, and their connectivity, are described in detail by Voogd and Barmack (2006). Secondly, only those connections relevant to the OKR are shown: thus, vestibular inputs are omitted. These simplifications are the ones usually made in linear-system modeling of the OKR (e.g., Carpenter, 1988).

In the circuit shown in Figure 3, the flocculus is placed to learn an internal model incorporating any structure that may be present in the world velocity input. This structure may be present either in the external world itself (exafference), for example water currents, or derive from the organism's own movements (reafference). Successful learning enables the floccular output to alter eye-movement commands, making them more effective in reducing retinal slip. This procedure has similarities with predictive noise cancellation, which acts to remove interference (noise) from sensory signals (e.g., Anderson et al., 2010, 2012; Porrill et al., 2013).

#### Time Delay, the Smith Predictor, and Internal Models

Time delay in feedback control loops creates a significant problem regarding stability (aside from the adaptation problem described above). The OKR is no exception to this due to the delay in visual processing of the retinal slip signal (St.-Cyr and Fender, 1969; Waespe and Henn, 1987). Therefore it is reasonable to question whether speculation on the action of the cerebellum as a Smith predictor is relevant to this study (Miall et al., 1993). Young and Robinson have separately proposed control schemes where the cerebellum acts as a forward model of oculomotor plant dynamics in smooth pursuit and OKR, implicitly constructing a control architecture that was closely related to the Smith predictor (Young, 1971; Robinson, 1977; Robinson et al., 1986). The approach we have taken here is to construct the adaptive model with no assumptions regarding the functional role of the cerebellum. On completion of learning we examined the dynamic behavior of the cerebellar model, investigating the internal model hypothesis (Wolpert et al., 1998) in the context of the OKR. We found that the cerebellar filter model did not resemble oculomotor plant dynamics so much as a generic lag compensator and that functionally such an internal oculomotor plant model did not logically fit into the control scheme (however, this is a simplified model so no definitive conclusions can be drawn). Instead we observed that the cerebellar filter acted to directly raise the gain of the closed loop system in the frequency region around 0.1 Hz and improve the phase response in the same region.

### Future Work

Ahrens et al. (2012) investigated the neural substrates of an optomotor behavior related to the OKR in larval zebrafish. The behavior involved swimming in the direction of translational optic flow, thereby stabilizing the fish's position in the presence of water currents. Motor output rapidly adapted to changes in visual input. This behavior was accompanied by neural activity in multiple brain regions including the cerebellum and inferior olive, and its adaptation prevented by lesions of the inferior olive. Portugues et al. (2014) subsequently extended this investigation to whole-brain mapping of the networks involved in rotational OKR, as modeled here. The circuits discovered resemble those described in mammals, include the cerebellum, and show little variation between individual fish. These studies suggest that the larval zebrafish's small and transparent brain offers the opportunity of further investigating the basic circuitry underlying OKR adaptation, and of exploring the neural mechanisms underlying the effects of stimulus predictability and the formation of internal models.

#### Summary

We have developed an adaptive model of the OKR based on the adaptive filter representation of cerebellar cortex proposed by Fujita (1982). The model demonstrates how the cerebellum improves the disturbance rejection characteristics in this exemplar problem of an adaptive feedback control task. The model describes behavioral data, specifically the step response of the OKR in primate. Our results have shown that the adaptation of the OKR is extremely sensitive to the world velocity signal used to excite the OKR feedback control loop. When the world velocity signal is a predictable single frequency sine wave the feedback control performance is much improved compared to an unpredictable colored noise signal. Finally, the nature of the world statistics were shown to be crucial in driving adaptation of the cerebellar model to an OKR loop that described experimental step response data.

## Data Availability Statement

The datasets generated for this study are available on request to the corresponding author.

## Author Contributions

SA implemented the computational simulations, analyzed the results, and co-wrote the manuscript. JP conceived the work and helped implement the computational simulations. PD conceived the work and co-wrote the manuscript.

### Conflict of Interest

The authors declare that the research was conducted in the absence of any commercial or financial relationships that could be construed as a potential conflict of interest.
